# Doxycycline post-exposure prophylaxis for sexually transmitted infections impacts the gut antimicrobial resistome

**DOI:** 10.21203/rs.3.rs-4243341/v1

**Published:** 2024-04-17

**Authors:** Charles Langelier, Victoria Chu, Abigail Glascock, Deborah Donnell, Cole Grabow, Clare Brown, Ryan Ward, Christina Love, Katrina Kalantar, Stephanie Cohen, Chase Cannon, Michael Woodworth, Colleen Kelley, Connie Celum, Anne Luetkemeyer

**Affiliations:** University of California, San Francisco; UCSF; Chan Zuckerberg Biohub; Fred Hutchinson; University of Washington; University of Washington; UCSF; UCSF; Chan Zuckerberg Initiative; University of California San Francisco; University of Washington; Emory University; Emory University School of Medicine; University of Washington; Zuckerberg San Francisco General Hospital UCSF

**Keywords:** antimicrobial resistance, antibiotic resistance, resistome, doxy-PEP, doxycycline, sexually transmitted infection, men who have sex with men

## Abstract

Doxycycline post-exposure prophylaxis (doxy-PEP) reduces bacterial sexually transmitted infections (STIs) among men who have sex with men and transgender women. While poised for widespread clinical implementation, the impact of doxy-PEP on antimicrobial resistance remains a primary concern as its effects on the gut microbiome and resistome, or the antimicrobial resistance genes (ARGs) present in the gut microbiome, are unknown. To investigate these effects, we studied participants from a randomized clinical trial who either received doxy-PEP as a one-time doxycycline 200 mg taken after condomless sex (DP arm, n = 100) or standard of care treatment (SOC arm, n = 50). From self-collected rectal swabs at enrollment (day-0) and after 6 months (month-6), we performed metagenomic DNA sequencing (DNA-seq) or metatranscriptomic RNA sequencing (RNA-seq). DNA-seq data was analyzable from 127 samples derived from 89 participants, and RNA-seq data from 86 samples derived from 70 participants. We compared the bacterial microbiome and resistome between the two study arms and over time. Tetracycline ARGs were detected in all day-0 DNA-seq samples and 85% of day-0 RNA-seq samples. The proportional mass of tetracycline ARGs in the resistome increased between day-0 and month-6 in DP participants from 46–51% in the metagenome (p = 0.02) and 4–15% in the metatranscriptome (p < 0.01), but no changes in other ARG classes were observed. Exposure to a higher number of doxycycline doses correlated with proportional enrichment of tetracycline ARGs in the metagenome (Spearman’s ρ = 0.23, p < 0.01) and metatranscriptome (Spearman’s ρ = 0.55, p < 0.01). Bacterial microbiome alpha diversity, beta diversity, and total bacterial mass did not differ between day-0 and month-6 samples from DP participants when assessed by either DNA-seq or RNA-seq. In an abundance-based correlation analysis, we observed an increase over time in the strength of the correlation between tetracycline ARGs and specific bacterial taxa, including some common human pathogens. In sum, doxy-PEP use over a 6-month period was associated with an increase in the proportion of tetracycline ARGs comprising the gut resistome, and an increase in the expression of tetracycline ARGs. Notably, doxy-PEP did not significantly alter alpha diversity or taxonomic composition of the gut microbiome, and did not demonstrate significant increases in non-tetracycline ARG classes. Further studies and population level surveillance are needed to understand the implications of these findings as doxy-PEP is implemented as a public health strategy.

## Introduction

Doxycycline post-exposure prophylaxis (doxy-PEP) is highly efficacious in preventing bacterial sexually transmitted infections (STIs) in randomized, controlled clinical trials among men who have sex with men (MSM) and transgender women living with HIV or on pre-exposure prophylaxis (PrEP) to prevent HIV infection^1–3^. This new public health strategy is being incorporated into preliminary guidelines for STI prevention with an initial Grade A1 recommendation to consider use of doxy-PEP for MSM and transgender women from the US Centers for Disease Control and Prevention^4^ and a World Health Organization guidelines^5^ in process. Widespread implementation of doxy-PEP among all MSM and transgender women could increase doxycycline consumption substantially, with high-end estimates of as much as 3.36 million doses per month in the U.S.^6^. As such, a primary outstanding concern is the potential for doxy-PEP to select for antimicrobial resistant bacteria and adversely impact the human microbiome^7–9^.

Antimicrobial resistance is a major global public health challenge that complicates the management of infectious diseases^10^. The overuse and misuse of antibiotics in both human healthcare and agriculture are major contributors to this problem^11–13^. In particular, multidrug resistant *Neisseria gonorrhoeae*, which can be resistant to all first line antimicrobial treatments, has been increasing in prevalence and has been labeled as an urgent threat by the U.S. CDC^14–18^. Given this, there are concerns that doxy-PEP implementation may lead to increased tetracycline-resistant *N. gonorrhoeae*, which may be less susceptible to doxy-PEP; to co-selection for beta-lacatam resistance in *N. gonorrhoeae*, which is currently the first-line antibiotic treatment; and to selection for antimicrobial resistance in commensal organisms as well as disease-causing pathogens such as *Staphylococcus aureus*.

The few studies that have evaluated the impact of doxycycline exposure on the human microbiome involved daily doxycycline use and were based primarily on bacterial culture or 16S rRNA gene amplicon sequencing, limiting their ability to evaluate the antimicrobial resistance genes (ARGs) in the the microbiome, termed the resistome^19–22^. In contrast, metagenomic DNA sequencing (DNA-seq) allows for comprehensive assessment of bacterial genomes and the genetic potential for antimicrobial resistance, while metatranscriptomic RNA sequencing (RNA-seq) provides a functional profile of actively transcribed genes, including ARGs.

To address the outstanding question of whether doxy-PEP impacts the ecology of the gut microbiome and resistome, we study longitudinally collected rectal swabs from DoxyPEP clinical trial participants using a combination of DNA and RNA sequencing approaches. We find that doxy-PEP use increases the proportion and expression of tetracycline resistance genes in the gut microbiome, while minimally affecting community composition and diversity. Together, our findings provide new insight into the microbiological impacts of doxy-PEP prior to its widespread deployment for STI prevention.

## Results

### Clinical cohort

We studied 100 doxy-PEP and 50 standard of care (SOC) participants from the 501 participants enrolled in the DoxyPEP clinical trial^2^, and performed DNA- and RNA-sequencing on rectal swabs self-collected at time of enrollment and after six months. We preferentially evaluated participants with the highest reported doxy-PEP use. Among the selected participants, 89 had analyzable DNA-seq samples (58 day-0 samples, 69 month-6 samples); 70 had analyzable RNA-seq samples (26 day-0 samples, 60 month-6 samples) (Supp. Figure 1). There were no significant differences between participants in the doxy-PEP arm versus the SOC arm with regards to age, race/ethnicity, education level, housing situation, or proportion of participants living with HIV (Supp. Table 1). Among the 69 participants with month-6 DNA-seq data, the median number of doxycycline doses taken since enrollment was zero (IQR: 0–7 doses) for the SOC arm and 50 (IQR: 27–64 doses) for the doxy-PEP arm (Supp. Table 2). Some participants in the SOC arm received doxycycline for clinical indications such as STI treatment. For the 60 participants with month-6 RNA-seq samples, the median number of doxycycline doses in the SOC arm was also zero (IQR: 0–7 doses), as compared with 42 (IQR: 29–65 doses) in the doxy-PEP arm (Supp. Table 2).

### Impact of doxy-PEP on the gut antimicrobial resistome

We first assessed the presence of tetracycline resistance genes in the resistome at enrollment. Among day-0 samples, a total of 41 tetracycline resistance genes were detected by DNA-seq and 17 tetracycline resistance genes by RNA-seq. Tetracycline ARGs were the most prevalent ARG class in the resistome with at least one tetracycline ARG detected in 100% (n = 58/58) of samples by DNA-seq and 85% (n = 22/26) of samples by RNA-seq (Supp. Figure 2A). Tetracycline ARGs represented the largest proportion (46%) of the resistome by mass (Supp. Figure 2B), but accounted for only 4% of the expressed resistome mass at the time of enrollment (Supp. Figure 2C).

**Figure 1:**
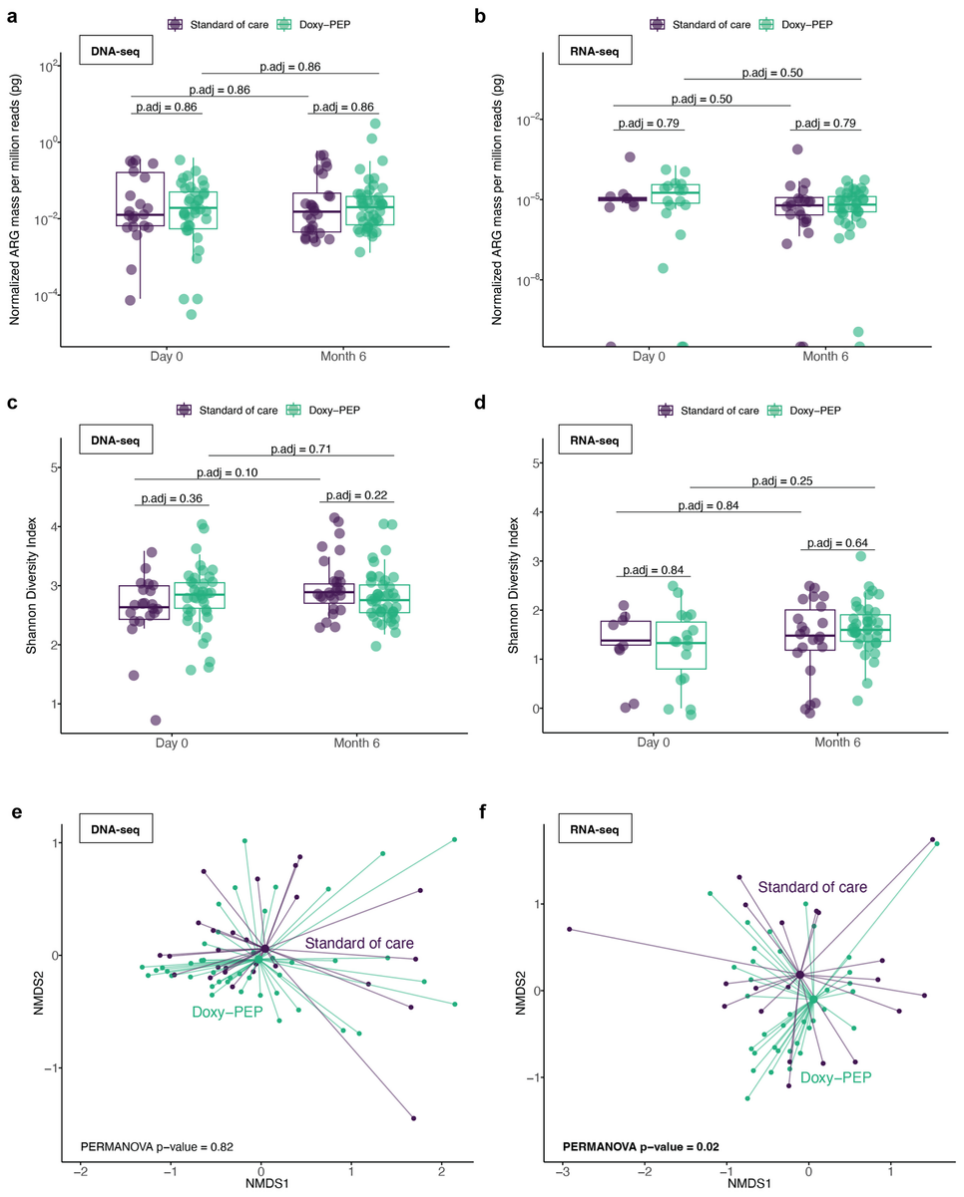
Impact of Doxy-PEP use on the gut resistome for DNA-seq samples (A, C, E) and RNA-seq samples (B, D, F). (A, B) Normalized resistome mass for doxy-PEP versus SOC participants (DNA-seq: n = 127, RNA-seq: n = 86). (C, D) Resistome alpha diversity (Shannon Diversity Index) in doxy-PEP versus SOC participants (DNA-seq: n = 127, RNA-seq: n = 86). (E, F) Resistome beta diversity (Bray-Curtis Index) in doxy-PEP versus SOC participants at six months (DNA-seq: n = 69, RNA-seq: n = 60). One outlier from the Doxy-PEP arm was omitted from the RNA-seq beta diversity plot for graphical purposes with coordinates of (NMDS1: 9.9, NMDS2: −0.3), but was included in the calculations. P-values were calculated using the Wilcoxon rank-sum test and adjusted for multiple comparisons (A-D). P-values for beta diversity was calculated using the PERMANOVA test and adjusted for multiple comparisons (E, F). Significant p-values (<0.05) are bolded. Abbreviations: ARG, antimicrobial resistance gene; p.adj, adjusted p-value; NMDS, non-metric multidimensional scaling.

We evaluated for ecological differences in the resistome between the doxy-PEP and SOC arms by assessing resistome: (1) mass, computationally estimated from spiked-in mass standards; (2) alpha diversity, measured by Shannon diversity index, and (3) beta diversity, measured by Bray-Curtis dissimilarity index. No differences between doxy-PEP and SOC arms were observed in resistome mass (Figs. 1a, 1b) or alpha diversity (Figs. 1c, 1d) at any time points by either DNA-seq or RNA-seq. While no differences in beta diversity were observed by DNA-seq (Fig. 1e), significant compositional differences in the expressed resistome were found between the doxy-PEP and SOC arms by RNA-seq at month-6 (p_adj_ = 0.02 by PERMANOVA, Fig. 1f).

**Figure 2:**
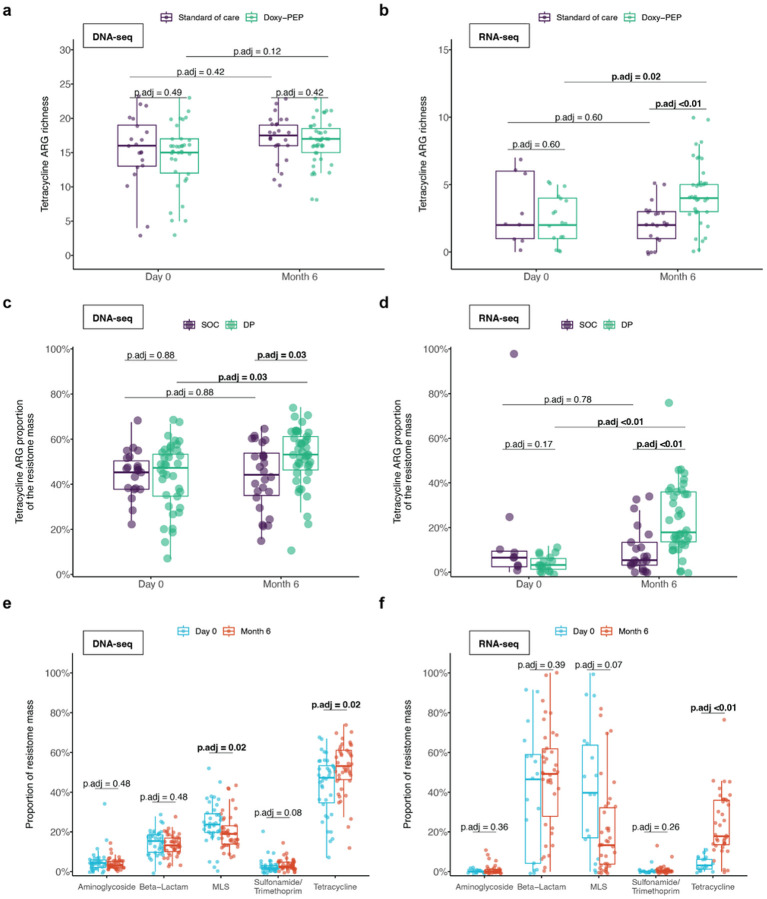
Impact of Doxy-PEP use on tetracycline and non-tetracycline ARGs for DNA-seq samples (A, C, E) and RNA-seq samples (B, D, F). (A, B) Tetracycline ARG richness and (C, D) tetracycline ARG proportion of resistome mass were compared between standard of care and doxy-PEP arms at each visit and over time (DNA-seq: n = 127, RNA-seq: n = 86). (E, F) The proportion of the resistome mass by ARG classes over time within the doxy-PEP arm (DNA-seq: n = 80, RNA-seq: n = 55). P-values were calculated using the Wilcoxon rank-sum test and adjusted for multiple comparisons. Significant p-values (<0.05) are bolded. Abbreviations: ARG, antimicrobial resistance gene; p.adj, adjusted p-value; MLS, macrolide-lincosamide-streptogramin.

We further evaluated the impact of doxy-PEP use on different ARG classes by comparing the day-0 and month-6 samples within the doxy-PEP arm. While tetracycline ARG richness was not found to differ over time by DNA-seq (p_adj_ = 0.12, Fig. 2a), the number of detectably expressed tetracycline ARGs increased by RNA-seq (p_adj_ = 0.02, Fig. 2b). Among participants in the doxy-PEP arm, the proportion of tetracycline ARGs in the resistome identified by DNA-seq increased over the 6-month study period (46–51%, p_adj_ = 0.02, Fig. 2c), as did the proportion of expressed tetracycline ARGs identified by RNA-seq (4–15%, p_adj_ <0.01, Fig. 2d). The most common mechanism of tetracycline resistance observed was ribosomal target protection in both the metagenome and metatranscriptome (Supp. Figures 3a, 3b).

No proportional increases were noted in other non-tetracycline ARG classes (Figs. 2e, 2f), suggesting specificity of doxy-PEP use for tetracycline ARGs. While no change in tetracycline ARG abundance was observed by DNA-seq over time, tetracycline ARG expression by RNA-seq significantly increased in doxy-PEP participants over six months of follow-up (Supp. Figure 4). A sensitivity analysis adjusting for HIV status demonstrated similar results (Supp. Table 3). We also evaluated for specific beta-lactam resistance genes of high public health concern at enrollment and at month-6. The extended-spectrum beta-lactamase-encoding gene, *CTX-M*, was detected at month-6 in one SOC participant by both DNA-seq and RNA-seq, and three doxy-PEP participants (one by DNA- and RNA-seq, one by DNA-seq only, and one by RNA-seq only); CTX-M was not detected in any day-0 samples by either DNA-seq or RNA-seq. The carbapenemase genes *KPC, NDM, VIM,* and OXA-48 were not detected in any samples by DNA-seq or RNA-seq.

**Figure 3:**
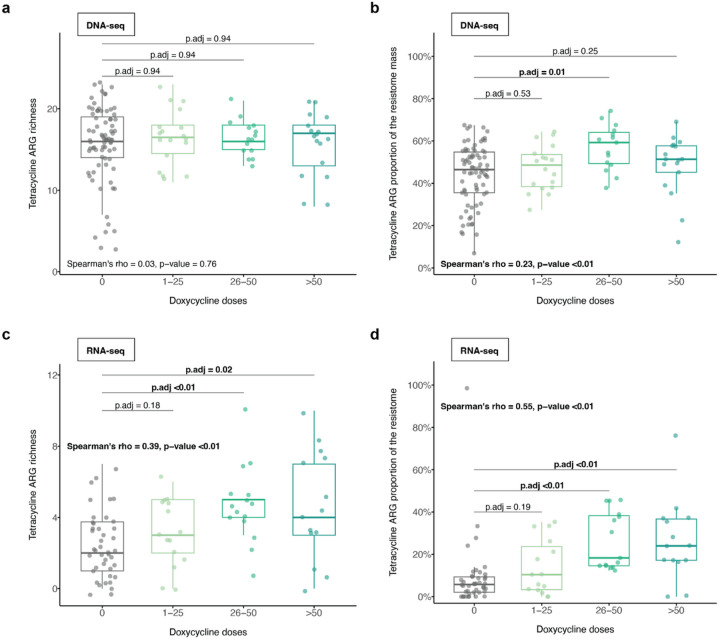
Impact of Doxy-PEP use on tetracycline ARGs by number of doxycycline doses received for DNA-seq samples (A, B) and RNA-seq samples (C, D). A test of trend was used to compare (A, C) tetracycline ARG richness and (B, D) proportion of tetracycline ARG mass to resistome mass by number of doxycycline doses received (DNA-seq: n = 127, RNA-seq: n = 86). P-values were calculated using the Wilcoxon rank-sum test and adjusted for multiple comparisons. The Spearman’s rank correlation test was used to calculate Spearman’s ρ and p-value. Significant p-values (<0.05) are bolded. Abbreviations: ARG, antimicrobial resistance gene; p.adj, adjusted p-value.

We next asked whether doxycycline influenced tetracycline ARGs in a dose-dependent manner. In the metagenome, the number of doxycycline doses was not associated with changes in richness of tetracycline ARGs (Spearman’s ρ = 0.03, p = 0.76, Fig. 3a). However, it was weakly positively correlated with the proportion of tetracycline ARGs in the resistome (Spearman’s ρ = 0.23, p < 0.01, Fig. 3b) potentially indicating preferential growth of tetracycline-ARG carrying bacteria. Furthermore, in the metatranscriptome, the number of doxycycline doses was strongly positively correlated with both tetracycline ARG richness (Spearman’s ρ = 0.39, p < 0.01) and the relative proportion of expressed tetracycline ARGs in the resistome (Spearman’s ρ = 0.55, p < 0.01, Figs. 3c, 3d). We noted that only participants who had reported taking > 25 doxycycline doses over the six month follow-up period demonstrated significantly increased tetracycline ARG richness and proportional tetracycline ARG representation compared with those who had not taken any doxycycline (Figs. 3c, 3d).

**Figure 4:**
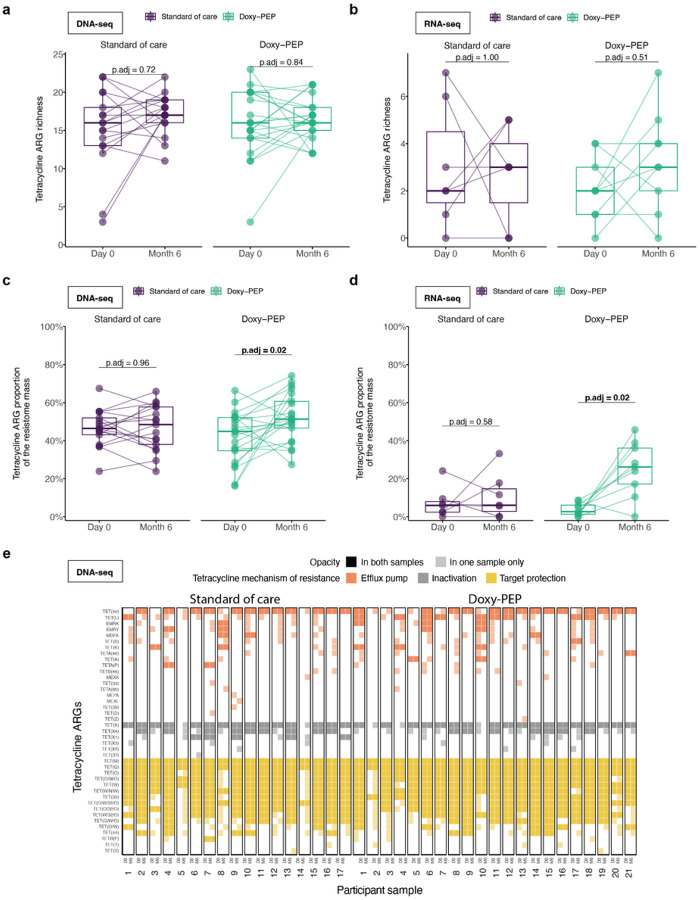
Impact of Doxy-PEP use on tetracycline ARGs in sets of paired DNA-seq samples (A, C, E) and paired RNA-seq samples (B, D). (A, B) Tetracycline ARG richness and (C, D) the proportion of tetracycline ARG mass to resistome mass were compared between standard of care and doxy-PEP arms at each visit and over time (DNA-seq: n = 38 sets of paired samples, RNA-seq: n = 16 sets of paired samples). (E) Heatmap of the tetracycline ARGs detected in DNA-seq data of paired samples (day-0 and month-6 samples) for the standard of care and Doxy-PEP arms (n = 38 sets of paired samples). P-values were calculated using the Wilcoxon signed-rank test for paired samples and adjusted for multiple comparisons. Significant p-values (<0.05) are bolded. Abbreviations: ARG, antimicrobial resistance gene; p.adj, adjusted p-value; D0, day-0; M6, month-6.

We performed a secondary analysis with paired samples (DNA-seq: 38 participants, RNA-seq: 16 participants) (Supp. Figure 1). Among the paired samples, no changes in tetracycline ARG richness were noted (Figs. 4a, 4b). In the doxy-PEP arm, however, we observed a significant increase in the proportion of tetracycline ARGs in the resistome when measured by either DNA-seq (45–51%, p_adj_ = 0.02) or RNA-seq (6–26%, p_adj_ = 0.02) (Figs. 4c, 4d). We noted that in both the SOC and doxy-PEP arms, tetracycline ARGs with diverse mechanisms of action were both lost and gained between the day-0 and the month-6 paired samples (Fig. 4e).

### Impact of doxy-PEP on the gut microbiome and metatranscriptome

**Figure 5:**
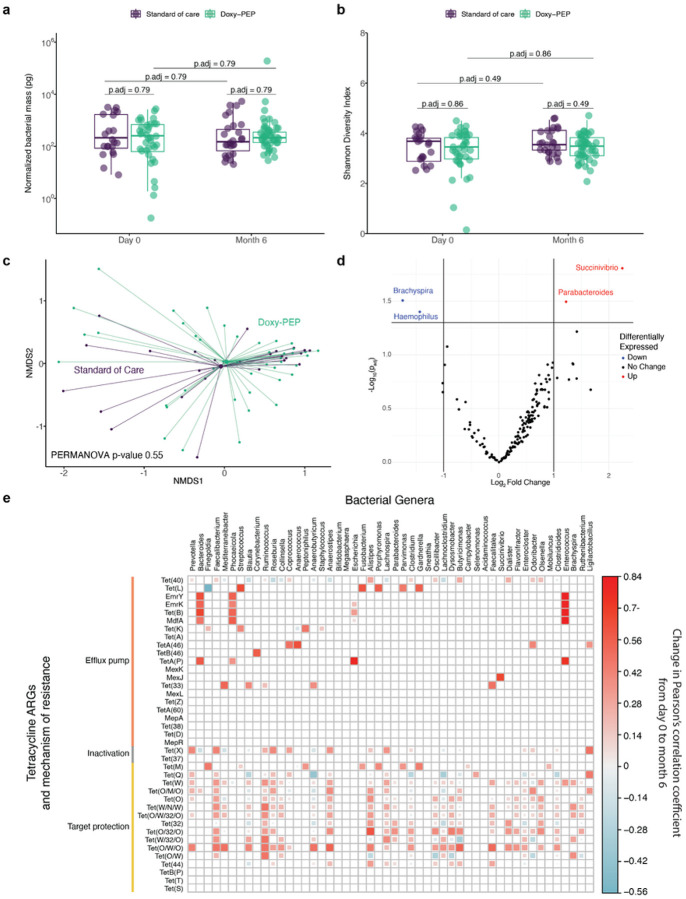
Bacterial microbiome metrics and correlation with tetracycline ARGs for DNA-seq samples. (A) Normalized mass of the bacterial microbiome and (B) alpha diversity measured by Shannon Diversity Index were compared between standard of care and doxy-PEP arms at each visit and over time (n = 127). (C) Beta diversity was compared between the SOC and doxy-PEP arms at the month-6 time point (n = 69). (D) A differential abundance analysis of bacterial genera was performed using DeSeq2 comparing doxy-PEP samples at day-0 and month-6 with a significance level of 0.05, adjusted for multiple comparisons (n = 80). (E) A plot demonstrating the change in Pearson’s correlation coefficient between tetracycline ARG abundance and bacterial genera abundance from day-0 to month-6 doxy-PEP samples for tetracycline ARGs and bacterial genera that were found to be significantly (p.adj <0.05, adjusted for multiple comparisons) correlated at month-6 in the doxy-PEP arm (n = 80). Color and size of the square filling represent the degree of change in the correlation coefficient. Blank squares represent cases where there was not a significantly correlation between the tetracycline ARG and the bacterial taxa at month 6 or there was not enough data to evaluate for correlation. P-values for mass, alpha diversity, and species of interest were calculated using the Wilcoxon signed-rank test and adjusted for multiple comparisons. The p-value for beta diversity was calculated using the PERMANOVA test and adjusted for multiple comparisons. Abbreviations: ARG, antimicrobial resistance gene; p.adj, adjusted p-value.

Having observed an impact of doxy-PEP on the resistome, we next evaluated the effects on gut microbial communities. We found no differences in normalized bacterial mass of the gut microbiome (Fig. 5a) or metatranscriptome (Supp. Figure 5a) between the doxy-PEP and SOC arms at day-0 or month-6, or within study arms between time points. In addition, no differences were observed in bacterial taxonomic alpha diversity between arms or time points (Fig. 5b). In the metatranscriptome, while we observed increased alpha diversity at month-6 in the SOC arm compared with the doxy-PEP arm (p_adj_ = 0.045), no differences in alpha diversity were observed at enrollment between arms or over time (Supp. Figure 5b). Finally, we tested for differences in microbial community composition, but found no differences in beta diversity between the SOC and doxy-PEP arms at the month-6 time point, or between day-0 and month-6 in the doxy-PEP arm (Fig. 5c, Supp. Figure 5c).

We next carried out a differential abundance analysis of bacterial taxa between enrollment and the month-6 timepoint in the doxy-PEP arm using DNA-seq data. Only four bacterial genera were significantly different between the two time points; we found an increase in the relative abundance of *Parabacteroides* and *Succinivibrio* and a decrease in *Brachyspira* and *Haemophilus* in the gut microbiome (Fig. 5d). We confirmed that no differences in the relative abundance of the well-known enteric and STI pathogens *C. difficile, N. gonorrheae* or *M. genitalium* existed between enrollment and month-6 in doxy-PEP participants (Supp. Figure 6). We did, however, observe a possible reduction in *C. trachomatis* abundance (p = 0.06, Supp. Figure 6).

### Correlations within the resistome and microbiome

To identify linkages between the abundances of tetracycline ARGs and bacterial taxa within the gut microbiome, we performed multi-dimensional correlation analysis of day-0 and month-6 DNA-seq samples from doxy-PEP participants (Supp. Figure 7). Significant positive correlations were found between many tetracycline ARGs and bacterial genera, both pathogenic and commensal at day-0 and month-6; no statistically significant negative correlations were noted. To understand how the strength of the correlations between tetracycline ARGs and bacterial taxa changed over time in the setting of doxy-PEP use, we plotted the change in Pearson’s correlation coefficient between day-0 and month-6 (Fig. 5E). Many well established commensal genera (e.g., *Faecalibacterium*) exhibited increased correlations with tetracycline target protection ARGs over time. In addition, the abundance of several potentially pathogenic genera (e.g., *Enterococcus, Streptococcus, Escherichia*) demonstrated a strong positive increase in the Pearson’s correlation coefficient from the day-0 to month-6 samples with respect to several tetracyline efflux pump ARGs.

## Discussion

In patients from a randomized, controlled trial^2^, doxy-PEP use over six months minimally affected the taxonomic composition of the gut bacterial microbiome. However, we found a significant expansion of tetracycline ARGs in the resistome and a dose-dependent increase in their active expression. Importantly, the impact of doxy-PEP was restricted to tetracycline class ARGs, without evidence of co-selection for genes conferring resistance to other antibiotic classes. The clinical implications of the tetracycline ARG expansion in the gut resistome requires further investigation.

A healthy gut microbiota is essential for host metabolism, immunity, and intestinal barrier function^23^. Disruptions in the gut microbiome can lead to growth of pathogenic or resistant organisms^24^, increased susceptibility to infection^25^, and increased risk of non-communicable diseases such as obesity and cardiovascular disease^26^. In this cohort, we found that doxy-PEP use over six months did not significantly alter gut microbiome alpha diversity, beta diversity, or mass. Despite stability of these community-level measures, differential abundance analysis demonstrated some taxonomic shifts over the six months of doxy-PEP use, including a decrease in *Brachyspira* and an increase in *Succinivibrio*, of uncertain significance. Our findings are consistent with two prior culture-based studies of long-term daily low-dose (20 mg twice daily) doxycycline use in humans, which demonstrated minimal changes in gut microbiota^21,22^. A recent metagenomic study evaluating the impact of long-term daily doxycycline exposure, however, found significant alterations in the composition of skin microbiota, with more varying effects on the oral and fecal communities^27^. Although we did not find substantial compositional gut microbiome shifts, it is possible that that other anatomical sites may have experienced more significant perturbations in the microbiomes.

Tetracycline ARGs were the most prevalent and abundant ARG class represented in the gut microbiome comprising 46% of ARG mass even prior to doxy-PEP exposure, a finding consistent with observations from worldwide population studies of the human gut microbiome^28,29^. The widespread prophylactic use of tetracyclines in livestock selects for tetracycline resistant organisms, and may contribute to the predominance of tetracycline ARGs in the human gut microbiome^30,31^, along with tetracycline use for treatment of STIs and other indications, which is expected to be common in this study population. Furthermore, tetracycline resistance among *Bacteroides* species, has increased from 30% to > 80% over the last 50 years, hypothesized to be driven by horizontal transfer of tetracycline ARGs within the gut microbiome^32^.

We found that intermittent doxy-PEP use led to a small (46–51%), but significant proportional expansion of tetracycline ARGs in the gut resistome. These findings are consistent with several studies demonstrating increases in both tetracycline resistant bacteria^21,22,27^ and tetracycline ARG abundance^19^ following daily doxycycline use. Potential explanations for this finding include selective pressure from doxycycline exposure driving either the gain of new tetracycline ARGs or the elimination of susceptible bacteria and expansion of tetracycline-resistant bacteria. Because we did not find that doxy-PEP use led to significant increases in tetracycline ARG richness or mass over the six-month study period, the observed increase in tetracycline ARG proportional representation likely reflects expansion of previously existing resistance genes and their associated bacteria, rather than the acquisition of new tetracycline ARGs.

Because multiple ARGs can be found together on the same plasmid, antibiotic exposure in some cases can co-select for resistance to multiple drug classes. The data from this cohort suggest that co-selection for resistance to multiple antibiotic classes did not readily occur in the setting of doxy-PEP. While we found that doxyPEP use led to a significant increase in both the active transcription of tetracycline ARGs and their proportional expansion in the resistome, other classes of ARGs, including specific ARGs of public health concern, were largely unaffected.

The impacts of doxy-PEP use were much more striking at the transcriptional level, and highlights the additional benefits of assessing the microbiome using both DNA-seq and RNA-seq. Specifically, we found a dose-dependent increase in both tetracycline ARG expression, and proportional representation of tetracycline ARGs in the metatranscriptome. Notably, we only observed significant impacts on the resistome in participants who took 25 or more doxycycline doses over six months. In the DoxyPEP clinical trial^2^, the intervention arm reported using a median of 24 doxycycline doses over six months. These findings are in line with a recent study evaluating the impact of low- and high-dose doxycycline regimens (20mg twice daily vs 100mg twice daily) on skin flora, which found that the higher dose regimen was associated with more emergence and selective expansion of doxycycline-resistant staphylococci on the skin^27^. Interestingly, doxycycline-resistant *S. epidermidis* isolates recovered from individuals receiving the low-dose regimen had lower minimum inhibitory concentrations of doxycycline compared to those recovered from individuals receiving the high-dose regimen, supporting the idea that dose and frequency^33^ of doxycycline exposure may contribute to the emergence of resistance.

The relationship between detection of tetracycline ARGs and phenotypic antimicrobial resistance is not well understood. The DoxyPEP trial^2^ found an absolute proportional increase in tetracycline-resistant *N. gonorrhoeae* infections at 12 months, although the overall number of isolates was small and statistical comparisons were not performed. In an effort to understand which bacteria were associated with the expansion in tetracycline ARGs, we performed abundance-based correlation analyses between tetracycline ARGs and bacterial genera in the gut microbiome. We found significant and increasing correlations between tetracycline efflux pump ARGs and abdundance of several genera encompassing clinically relevant human pathogens including *Enterococcus, Streptococcus*, and *Escherichia* in doxy-PEP participants after six months of doxycycline use. These results suggest that at least a proportion of the increased tetracycline ARG mass in the resistome may be associated with potential bacterial pathogens.

In addition, we observed abundance-based correlations between tetracycline ARGs and enteric commensals, with correlations increasing in strength from day-0 to month-6 in the doxy-PEP arm. The gut microbiome is a well-known reservoir of ARGs^34^ that facilitates horizontal gene transfer between commensal and pathogenic bacteria^35,36^. This is of particular concern as many tetracycline ARGs are associated with plasmids, transposons and other mobile genetic elements^37^, which could facilitate their transfer to pathogenic bacteria carried in the gut microbiome. Further studies using long read sequencing or high-throughput chromosome conformation capture (Hi-C) metagenomic sequencing^38^ is needed to definitively assess connections between specific bacterial taxa and tetracycline ARGs in the setting of doxy-PEP.

Strengths of this study include leveraging a clinical trial to carry out the first in-depth assessment of doxycycline post-exposure prophylaxis use on the gut microbiome and resistome, detailed information on participant-reported doxycycline use enabling dose-response analyses, and combining metagenomics and metatranscriptomics to assess both the presence and active transcription of microbes and their ARGs. Furthermore, to our knowledge, this is the largest antimicrobial resistome study to date evaluating the impacts of doxycycline, a widely used broad spectrum antibiotic for the treatment and prophylaxis of human and animal infectious diseases.

We also acknowledge the limitations of this study. First, samples represented only a subset of the DoxyPEP clinical trial participants over the first 6 months of follow-up, and, in the doxy-PEP, samples from those with higher doxy-PEP use were preferentially selected, which may have biased findings away from the null compared with the average individual using doxy-PEP. Second, some participants from the SOC arm received doxycycline for STI treatment or other clinical indications, which may have biased the findings towards the null. Third, our analyses were limited by the quality of the self-collected rectal swabs; many specimens did not meet the minimum nucleic acid or sequencing quality standards and were excluded from the analysis. This reduction in sample size may have obscured subtle changes in the resistome or microbiome. Fourth, we did not have data on the exact timing of the doxy-PEP doses with respect to the rectal swab sample collection or longer term follow up samples to determine time to normalization of the microbiome and resistome. Fifth, we only evaluated the gut microbiome and doxy-PEP use may impact other microbiome sites such as skin and nasopharynx differently. Sixth, our specimens are limited to six months of follow-up; longer term data are needed to understand doxy-PEP impact with more extended use. Finally, as we used short read Illumina sequencing, we were unable to definitively link tetracycline ARGs to specific bacterial species and thus had to rely on abundance correlation analyses as a proxy.

In sum, we found that doxy-PEP use increased both the relative proportion and expression of tetracycline ARGs, while minimally impacting the ecology of the gut microbiome. These findings contribute to our understanding of the ecological impacts of doxy-PEP on the human gut microbiome and antimicrobial resistome, which at baseline is enriched in tetracycline ARGs. Further investigations are needed to explore the clinical implications of our findings, including population-based surveillance to monitor for emergence of tetracycline-resistant pathogens as doxy-PEP is more widely implemented in eligible populations.

## Methods

### Study design, clinical cohort, and ethics statement

The DoxyPEP trial^2^ compared doxy-PEP use (doxycycline post-exposure prophylaxis) to standard of care (no post-exposure prophylaxis) for 501 participants. The study was conducted at two HIV clinics and two sexual health clinics in San Francisco and Seattle. Individuals were eligible for enrollment if they were at least 18 years of age; male sex at birth; received a diagnosis of HIV or were on HIV PrEP; and had received a bacterial STI diagnosis of gonorrhea, chlamydia, or early syphilis in the previous 12 months. Participants were randomized in a 2:1 ratio to the doxy-PEP arm or the SOC arm. Participants in the doxy-PEP arm were counseled to take a 200 mg doxycycline hyclate dose within 72 hours after condomless anogenital, vaginal, or oral sex, and no more than one dose every 24 hours. Participants in both arms self-collected rectal swabs at enrollment (day-0) and at a 6-month visit (month-6). The study protocol^2^ was approved by the University of California, San Francisco institutional review board, which served as the primary institutional review board. All participants provided written informed consent.

For this analysis, a subset of 150 participants from the 510 DoxyPEP trial participants was selected for metagenomic sequencing of self-collected rectal swab samples. The 150 participants were selected based on the following criteria: 1) study arm group (50 SOC, 100 doxy-PEP), 2) HIV infection status (1:1 of participants living with HIV and participants on HIV PrEP), and 3) availability of both day-0 and month-6 rectal samples (Supp. Figure 1). The SOC participants were a simple random sample, while the doxy-PEP participants were the top 50 participants, including both persons with and without HIV infection, with the highest reported combined doxy-PEP use on the month-3 and month-6 study visits.

### Cohort description

We performed descriptive analysis of participant demographics and compared the participants in the doxy-PEP arm with the SOC arm. P-values for categorical variables were obtained using the Pearson’s chi-square test and Fisher’s exact test if counts were < 5; p-values for continuous variables were calculated using the Wilcoxon rank-sum test.

### Metagenomic sequencing

Metagenomic sequencing of DNA and RNA (DNA-seq, RNA-seq) was performed on the day-0 and month-6 rectal swabs from the 150 participants. Swabs were self-collected into DNA/RNA Shield collection tubes (Zymo Research) and stored at −80C within 2 weeks of collection. Total nucleic acid was extracted from 500 uL of DNA/RNA Shield solution using a previously described modified cetyltrimethylammonium bromide (CTAB)-based protocol^39^, and in samples with sufficient yield, normalized to 10ng total input per sample.

DNA-seq was carried out using the NEBNext Ultra II DNA Kit. Prior to RNA-seq, human cytosolic and mitochondrial ribosomal RNA was depleted using FastSelect (Qiagen). RNA was then fragmented and underwent library preparation using the NEBNext Ultra II RNA-seq Kit (New England Biolabs) according to manufacturer’s instructions. Both DNA-seq and RNA-seq library prep protocols were optimized for a LabCyte Echo acoustic liquid handler^40^. Finished libraries underwent paired-end Illumina sequencing on a NovaSeq 6000 instrument.

For the purposes of background contamination correction and to enable estimation of microbial mass, negative water controls and positive controls (spike-in RNA standards from the External RNA Controls Consortium (ERCC), ThermoFisher)^41^ were included in every RNA sample prior to RNA-seq library preparation. Reverse-transcribed complementary DNA ERCC standards were spiked into every DNA sample prior to DNA-seq library preparation.

### Initial detection of microbes and antimicrobial resistance genes

We leveraged the open-source CZ ID pipeline (https://czid.org/) as a first step to detect both microbes (mNGS pipeline v8.1) and ARGs (AMR pipeline v1.2.15)^42^. For microbial detection, the CZ ID pipeline performed subtractive alignment of the human genome (NCBI) from input raw fastq files, followed by quality and complexity filtering. The remaining microbial reads were then identified by an assembly-based alignment against reference genomes from the NCBI nucleotide (NT) database. Following background correction (see below), all remaining taxa with at least 10 hits to the NCBI NT database and 1 hit to the NCBI non-redundant protein database (NR) with a minimum alignment length of 50 bases were retained for downstream microbiome analyses. All samples with > 100,000 reads and DNA-seq samples with a duplicate compression ratio < 10 were retained for downstream analyses of microbes and ARGs. CZ ID’s antimicrobial resistance pipeline implements the Comprehensive Antibiotic Resistance Database (CARD)^43,44^ Resistance Gene Identifier (RGI) tool, which aligns quality-controlled reads against a continually updated and curated reference database of ARG sequences. ARGs with > 5% read coverage depth were retained for downstream analyses.

### Identification and mitigation of environmental contaminants

Negative water controls were processed in parallel with the participant samples for microbial and ARG detection, allowing for an estimation of the number of background reads expected for each taxon and ARG^40^. A negative binomial model was used to identify and select for taxa and ARGs present in the participant samples at an abundance significantly greater than in the negative controls^45^. The number of background reads were modeled as a negative binomial distribution, with mean and dispersion fitted on the negative controls. For each batch (DNA-seq only) and taxon/ARG, the mean parameter of the negative binomial was estimated by averaging the read counts across all negative controls. Using the functions glm.nb() and theta.md() from the R package MASS^46^ (v7.3.58.1), a single dispersion parameter across all taxa was then estimated. Taxa associated with a p-value of ≥ 0.05 were excluded; p-values were adjusted for multiple comparisons using the Benjamini-Hochberg False Discovery Rate method.

### Mass calculations

Microbial and ARG mass was calculated based on the total reads aligning to the ERCC RNA standards^41^ spiked into each sample (RNA-seq) or reverse-transcribed cDNA ERCC standards (DNA-seq). ERCC input mass was 25pg for DNA-seq samples and 2.5pg for RNA-seq samples. The following equations were utilized for microbial input mass, normalized by total million sequencing reads to account for sample variation in input mass:

$$\text{microbialinputmass}=\frac{\frac{\text{microbialreads}*\text{ERCCinputmass}}{\text{ERCCreads}}}{\text{sequencingreads }\left(\text{millions}\right)}$$

and for ARG input mass, normalized by total million sequencing reads:

$$\text{ARGinputmass}=\frac{\frac{\text{ARGdepth}*\text{ERCCinputmass}}{\text{ERCCreads}}}{\text{sequencingreads }\left(\text{millions}\right)}$$

ARG depth was defined as the mean read depth across the references sequence. The mass of an ARG class was the summation of the mass of all ARGs belonging to the class of interest. Similarly, total microbial or ARG mass of each sample was a summation of the mass of all microbes or ARGs, respectively.

### Statistical analyses

#### Resistome analysis

We evaluated the impact of doxy-PEP use on ecological parameters, including the resistome alpha diversity, resistome beta diversity, and total resistome mass. Alpha diversity was calculated by Shannon diversity index, accounting for ARG abundance (dpm) and evenness. Beta diversity was calculated using Bray-Curtis dissimilarity with 1000 permutations, accounting for presence/absence and abundance of the ARGs. Analysis of multivariate homogeneity of group dispersions was performed using the functions betadisper() and permutest(). Beta diversity was displayed via non-metric Multi-Dimensional Scaling (NMDS) and the function metaMDS(). The adonis2() function was used to perform a PERMANOVA test and adjusted for multiple comparisons. Both diversity calculations were performed using the R package “vegan” (v.2.6.4)^47^. Total resistome mass which was normalized using a log10 transformation.

We assessed the impact of doxy-PEP use on tetracycline ARG richness (number of distinct ARG types) and proportion of each ARG class mass to the total resistome mass. We focused on ARG classes where the median proportion of the ARG class mass of the resistome mass per sample was > 1% in any of the following subgroups (SOC day-0, SOC month-6, doxy-PEP day-0, doxy-PEP month-6) for DNA- or RNA-seq data; these ARG classes included aminoglycosides, beta-lactams, MLS, sulfonamide/trimethoprim, and tetracyclines (Supp. Table 4). ARGs that included tetracycline resistance but also conferred resistance to multiple other classes were “multi-drug efflux pumps”; these were not included in the ARG class analysis given the proportional mass was < 1% of the resistome mass (Supp. Table 4). We used linear regression models to examine the association between doxy-PEP use and the proportion of ARG class mass within the resistome, by ARG class while accounting for HIV infection status. We also compared ARG class abundance and expression; both were measured and normalized per million reads sequenced and gene length (depth per million, dpm) in the metagenome and the metatranscriptome, respectively. Within the tetracycline ARGs, we described the different mechanisms of resistance (tetracycline target protection, tetracycline inactivation, and tetracycline-specific efflux pumps) detected.

We evaluated whether there was a dose-dependent relationship between the number of reported doxycycline doses taken since enrollment and changes in the resistome. We considered a prophylactic dose (doxycycline 200 mg one time) as a single dose. For patients receiving doxycycline for STI treatment (doxycycline 100 mg twice a day for seven days), we considered a treatment day to be equivalent to a single prophylactic dose. The number of doxycycline doses was categorized as: 0 doses, 1–25 doses, 26–50 doses, and ≥ 50 doses. Spearman’s ρ test of trend (cor.test) from the R package “stats” (v4.2.1) was performed across these ordinal doxycycline dose categories for tetracycline ARG richness and proportion of tetracycline ARG to the resistome mass.

A sub-analysis of paired samples was performed to evaluate the impact of doxy-PEP use on tetracycline ARG richness and tetracycline ARG proportion of the resistome mass. P-values were calculated using the Wilcoxon signed-rank test for paired samples (wilcox_test, paired = TRUE) from the R package “rstatix” (v0.7.2). For all non-paired comparison tests, p-values were obtained by the Wilcoxon rank-sum test (wilcox_test, paired = FALSE).

#### Microbiome analysis

To examine the effect of doxy-PEP use on the global microbiome taxonomic composition, we analyzed the normalized and transformed mass of the bacterial components of the microbiome. We also examined differences in diversity metrics of the microbiome between the two arms at both time points and between time points within arms. Bacterial alpha diversity was calculated using the Shannon diversity index, accounting for bacterial abundance (nt rpm) and evenness. Bacterial beta diversity was calculated using Bray-Curtis dissimilarity in a similar manner to the resistome analysis, substituting bacterial abundance by nt rpm, with the R package “vegan” (v.2.6.4)^47^. To examine changes at a more granular level, we performed differential abundance analysis at both the genus and species level, adjusted for multiple comparisons, using R package “DESeq2” (v1.36.0). Specific species of interest, including common sexually-transmitted organisms, were also analyzed for differential abundance between day-0 and month-6 in the doxy-PEP arm using the Wilcoxon rank-sum test.

### Microbiome and ARG correlation

To identify microbial taxa associated with tetracycline ARGs, Pearson’s correlation analyses in doxy-PEP day-0 and month-6 samples were performed using the functions cor() and cor_pmat() from R package “rstatix” (v0.7.2). The correlation analyses were between the abundance (DNA-seq) or expression (RNA-seq) of tetracycline ARGs (dpm) and microbial taxa (rpm). Correlation analyses were adjusted for multiple comparisons. These analyses were performed at genus level, comparing the 50 most abundant bacterial taxa in combination with tetracycline resistance genes. To evaluate the change in the strength of correlations between tetracycline ARGs and bacterial taxa over time with doxy-PEP use, we calculated the change in the Pearson’s correlation coefficient (PCC) from day-0 to month-6 (ΔPCC = PCC_month6_ – PCC_day−0_).

All analyses were conducted in R (v4.2.1) and performed for both DNA-seq and RNA-seq data. All adjustments for multiple comparisons were by the Benjamini-Hochberg False Discovery Rate method. Figures were made using the following R packages “ggplot2” (v3.4.0) and “scales” (v1.2.1).
